# Supplementary material for: miR-142 Targets TIM-1 in Human Endothelial Cells: Potential Implications for Stroke, COVID-19, Zika, Ebola, Dengue, and Other Viral Infections
Source: Int J Mol Sci. 2022 Sep 6;23(18):10242. doi: 10.3390/ijms231810242 (PMC9499484; doi:10.3390/ijms231810242)
Supplement: Supplementary file 1 [file ijms-23-10242-s001.zip › ijms-1884390-supplementary.pdf]

Supplementary Figures

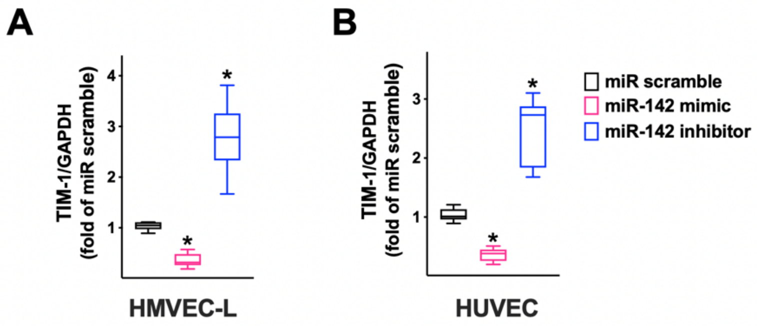

Figure S1. TIM-1 expression in two different types of human endothelial cells is reduced by miR-142 and increased by miR-142 inhibitor.

TIM-1 mRNA levels were measured by RT-qPCR in adult human lung microvascular endothelial cells (HMVEC-L, **A**) and human umbilical vein endothelial cells (HUVEC, **B**). The cells were transfected with miR-142 mimic, inhibitor, or scramble (negative control) for 48 h, normalizing to glyceraldehyde 3-phosphate dehydrogenase (GAPDH). All experiments were performed at least in triplicate; the box-and-whiskers graphs show the median and the 5th-95th percentiles; *:p<0.01 *vs* miR-scramble. Sequences of oligonucleotide primers are reported in Table 1.

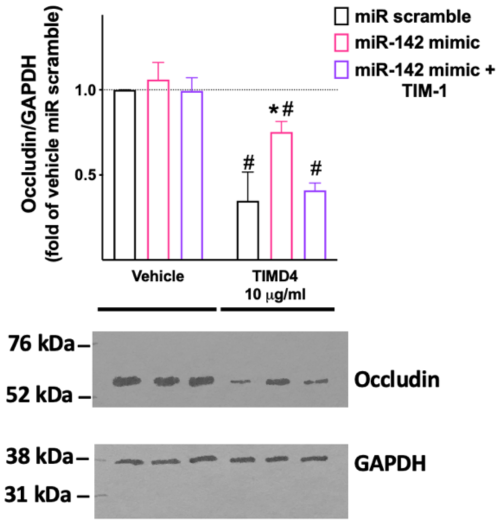

Figure S2. Occludin expression is regulated by miR-142.

Occludin protein levels were measured by Western blot in human brain microvascular endothelial cells (hBMECs) transfected with miR-142 mimic, miR scramble, or combining miR-142 mimic and TIM-1 overexpression; cells were treated for 48h with 10 ng/mL TIMD4 (TIM-1 agonist) or vehicle; representative immunoblot from triplicate experiments; *:p<0.01 vs miR-scramble, #:p<0.01 vs vehicle.
